# Ultrasound transducer disinfection in emergency medicine practice

**DOI:** 10.1186/s13756-016-0110-y

**Published:** 2016-04-04

**Authors:** Riley Hoyer, Srikar Adhikari, Richard Amini

**Affiliations:** College of Medicine, The University of Arizona, Tucson, AZ USA; Department of Emergency Medicine, The University of Arizona, PO Box 245057, Tucson, AZ 85724-5057 USA

## Abstract

**Background:**

External ultrasound transducer disinfection is common practice in medicine. Unfortunately, clinically significant organisms, such as methicillin-resistant *Staphylococcus aureus* (MRSA), *Pseudomonas aeruginosa,* and *Klebsiella pneumonia* spread throughout healthcare facilities via direct contact despite disinfection protocols. Ultrasound transducers and coupling gel provide potential vectors for pathogen transmission, especially in immunocompromised and high-risk patient populations. Our objective was to conduct a survey to investigate the variety of cleaning solutions or sanitary wipes used and evaluate current standard practice for transducer disinfection across emergency medicine training programs in the United States.

**Findings:**

Eighty-three academic emergency medicine programs participated in this study. Eighty-seven percent (95 % CI 80–94 %) of responding programs do not have a mandated protocol or standard contact time for transducer disinfection. Ninety percent (95 % CI 84–96 %) of institutions use disinfectant solution or disinfectant wipes, as the standard of practice, to cleanse ultrasound transducers after every use.

**Conclusions:**

Currently, there is a great deal of variability with regard to non-endocavitary transducer disinfection protocols that seems to stem from the vast number of disinfectant products and ultrasound manufacturer disparate recommendations. In order to mitigate risk to patients and reduce health care costs linked to nosocomial infections; healthcare providers, ultrasound companies, and disinfectant manufacturers must develop a universal use disinfectant and a standard protocol for ultrasound device disinfection for noncritical device disinfection in the emergency department.

**Electronic supplementary material:**

The online version of this article (doi:10.1186/s13756-016-0110-y) contains supplementary material, which is available to authorized users.

## Introduction

External ultrasound (US) transducer disinfection is common practice in modern medicine. With the rising utility of US as a diagnostic procedure in the emergency room setting, patient contact with ultrasound transducers has clearly increased over recent years. Currently, no universal standard for external-use US transducer disinfection exists. Clinically significant organisms, such as methicillin-resistant *S. aureus* (MRSA), *P. aeruginosa, K. pneumonia* spread throughout healthcare facilities via direct contact despite disinfection protocols [1]. US transducers and coupling gel provide potential vectors for pathogen transmission, especially in immunocompromised and high-risk patient populations [2]. In order to reduce nosocomial infection and improve disinfection practices, health-care providers must direct their attention to the lack of uniformity in US transducer disinfection.

The Center for Disease Control and Prevention (CDC) classifies all non-endocavitary US transducers as noncritical devices by definition meaning this medical equipment does not directly contact mucosal surfaces or non-intact skin [3]. Non-endocavitary US transducers do not require high-level disinfection and/or protection of the transducer with sterile transducer-cover between uses to prevent bacterial contamination. However, these devices require low-level disinfection. According to the CDC, low-level disinfection consists of greater than 1-minute exposure time of device to the following solutions or wipes: ethyl alcohol (70–90 %), sodium hypochlorite (5.25–6.15 %), phenolic germicidal detergent, iodophor germicidal detergent, or quaternary ammonium germicidal solution [4]. A variety of germicidal techniques have been studied as low-level disinfection including the following: >80 °C H_2_O, 0.5 % accelerated hydrogen peroxide, and quaternary ammonia germicidal wipes [5, 6]. In comparison, the CDC definition of high-level disinfection consists of 12–30 min of exposure time at >20 °C with the following solutions: gultaraldehyde-based formulations (>2 %), Ortho-phthalaldehyde (0.55 %), and hydrogen peroxide (7.5 %) [4].

After each use of an external ultrasound transducer, most institutions focus on two components of transducer disinfection: gross decontamination by removal of ultrasound gel, and low-level disinfection with germicidal wipes. Unfortunately, chemical components and recommended contact time vary between products. Commonly used germicidal wipes for US transducer disinfection include Super Sani-Cloth (isopropyl alcohol 55 %, aklyl ammonium compound 0.25 %), Sani-Cloth Plus (isopropanolol 14.85 %, quaternary ammonium 0.125 %), and Sani-Cloth HB (benzyl ammonium chlorides 0.07 %, quaternary ammonium 0.07 %). The Sani-Cloth manufacturer recommends a 2 min contact time for Super Sani-Cloth, 3 min contact time for Sani-Cloth Plus, and a 10 min contact time for Sani-Cloth HB [7–9]. Our objective was to conduct a survey to investigate the variety of cleaning solutions or sanitary wipes used and evaluate current standard practice for transducer disinfection across emergency medicine training programs in the United States.

## Methods

This was a cross-sectional survey study conducted electronically using a questionnaire developed by the investigators. The study was approved per the university’s process for approval of quality improvement projects involving humans. Respondents were from the Society for Academic Emergency Medicine (SAEM) ultrasound interest listserv. This listserv is comprised of ultrasound directors at academic emergency medicine programs primarily across the United States. A 5-item questionnaire (Additional file 1) on use of ultrasound transducer disinfection in the emergency department was created based on review of existing literature, knowledge of current practices, and discussions with experts in the field. Two emergency medicine (EM) physicians with expertise in point of care ultrasound reviewed the questionnaire for content validity. They evaluated all questions for relevance and clarity, as well as overall comprehensiveness of the questionnaire. The survey consisted of multiple-choice and free-text response questions regarding transducer disinfection including: current standard of practice in transducer disinfection, different disinfectant products, and duration of contact time between the transducer and disinfectant. The questionnaire was distributed via e-mail through the SAEM listserv. The e-mail message included a link to the survey (Surveymonkey.com, Palo Alto, California, USA), as well as an opt-out option. The survey was collected anonymously. Descriptive statistics was used to summarize the data using statistical analysis software (SAS) version 9.3 (Copyright, SAS Institute Inc., Cary, NC, USA). The survey responses are reported as the percentages of total respondents along with 95 % confidence intervals.

## Results

Eighty-three programs participated in this study. Eighty-seven percent (95 % CI 80–94 %) of responding programs do not have a standard of practice for transducer contact time with disinfectant. At 90 % (95 % CI 84–96 %) of the institutions, it is standard practice to use disinfectant solution or disinfectant wipes to cleanse ultrasound transducers after every use. Furthermore, 76 % (95 % CI 67–85 %) of the institutions performed a brief wipe (less than 15 s) as standard of practice for the recommended duration of contact time between the transducer and disinfectant. Only a small number of the surveyed institutions reported extended contact times for transducer disinfection: 9.6 % (95 % CI 3–16 %) 1 min, 2.4 % (95 % CI 1–6 %) 2 min, 2.4 % (95 % CI 1–6 %) 3 min. For the purpose of ultrasound transducer disinfection, EM residency programs use a variety of products, which are summarized in Table 1. The most commonly used product was PDI Super Sani-Cloth wipes 33 % (95 % CI 23–43 %).

**Table 1:** Most commonly used disinfectant wipes and solution at different emergency departments

Disinfectant	# of programs	Percentage^a^ (95 % CI)
PDI Super Sani-Cloth Wipe	27	33 % (23–43%CI)
PDI Sani-Cloth Plus Wipe	19	23 % (14–32%CI)
T-Spray Solution	15	18 % (10–26%CI)
PDI Sani-Cloth HB Wipe	14	17 % (9–25%CI)
Other^b^	15	18 % (10–26%CI)

^a^Total number of participating programs *N* = 83. ^b^Including: Sani-Cloth Bleach wipe, Sani-Cloth Hands wipe, Oxivir Tb wipe, Transeptic solution, Sani-Cloth AF3 wipe, Isopropyl alcohol wipe, and CaviWipes

## Discussion

Hospital-acquired infections in the United States present a significant risk for patient safety and place immense financial impact on the healthcare system. According to the Center for Disease Dynamics, Economics, and Policy (CDDEP), the United States spends 9.8 billion dollars on hospital-acquired infections annually [10]. Ultrasound transducers and coupling gel have the potential to spread hospital-acquired infections. Previous studies identified clinically significant organisms on ultrasound equipment and have implied that nocosomial infections can be spread by ultrasound equipment. One study demonstrated that 18 % of ultrasound transducers were contaminated with clinically significant organisms including: Enterococci, *S. aureus, Proteus mirabilis, Escherichia coli,* Group B Streptococci, and *Proteus vulgaris* [11]. Another study reported *K. penumoniae,* resistant to third generation cephalosporins, contaminated ultrasound coupling gel and infected both adult female and neonatal patients in an obstetrics and gynecology department [12]. In 2013, Chittick et al. discovered *P. aeurginosa,* in ultrasound gel, as the source of infection in patients who received a transthoracic echocardiogram during hospitalization [13].

The probability of potential infection caused by ultrasound transducers varies in the literature. One study assessed transducer disinfection across all departments at a military academic institution and found that there was an insufficient number of transducers grossly contaminated to yield valid results [14]. In contrast, Sanz et al. reported that gross debris was removed from ultrasound transducers after use in only 58 % of medical cases and only 33 % of trauma cases [15]. Gross contaminants must be wiped away prior to utilizing a cleaning solution or germicidal wipe because contents of the ultrasound gel may inhibit the germicidal effects of the disinfectant. After removing gross contamination, a germicidal agent should be used to disinfect the transducer, transducer cable, and US machine. In our study, 90 % of programs have standard practice for the use of germicidal wipes or solution for transducer disinfection after every use. Unfortunately, many of the programs adhere poorly to manufacturer recommended disinfectant contact times, with 76 % of programs performing only a brief wipe (less than 15 s) for transducer disinfection. Our data demonstrates that the most utilized germicidal wipe across academic institutions with EM residency programs in the United States is the PDI Super Sani-Cloth (33 % of programs). According to the manufacturer material safety data sheet (MSDS), the Super Sani-Cloth provides the greatest utility in the acute care setting as it requires a contact time of only 2 min. In addition, it provides a broader spectrum of antimicrobial coverage than Sani-Cloth Plus germicidal wipes [8]. Our survey revealed that, only 15 % of programs use at least a 1 min contact time as standard of practice for disinfection.

Unfortunately, manufacturers of commonly used ultrasound machines, the Philips Sparq and Zonare Z.one_pro_, do not recommend disinfection with PDI Super Sani-Cloth wipes [16, 17]. The active chemicals for disinfection in PDI Super Sani-Cloth wipes can be damaging to ultrasound transducer surfaces and device surfaces including transducer footprint, screen, cables, and connectors. Comparing the utility of PDI Sani-Cloth Plus, T-Spray and PDI Sani-Cloth HB, both T-spray and PDI Sani-Cloth HB are recommended for transducer, cable and connector disinfection; while, PDI Sani-Cloth PLUS should only be used to disinfect the transducers. Our review of germicidal wipes and solutions demonstrates that, of the disinfectants reported in our survey, Sani-Cloth HB provides the greatest utility of low-level disinfectants because these products are recommended by Phillips to disinfect all ultrasound surfaces including monitors and screens and this disinfectant is effective against 100+ microrganisms. [16] However, the 10 min recommended contact time of Sani-Cloth HB creates a challenge for the use of this wipe in a busy emergency department [9]. For disinfectants that require extended contact times, such as Sani-Cloth HB, providers should be aware that reduced contact times, shorter than recommended by the manufacturer, can result in ineffective disinfection and spread of clinically significant microorganisms in the hospital setting. Further investigation should be focused on collaborations between ultrasound companies and disinfectant wipe manufacturers in order to establish a universal disinfectant wipe that can be used on all ultrasound machine components that is fast acting and has broad spectrum germicidal properties.

### Limitations

This survey-based study has several limitations. Responder bias may have resulted in overrepresentation of programs with greater interest in ultrasound and perhaps greater expertise in transducer disinfection. Although the survey was pilot-tested, it was not validated; and a majority of the questions were closed-ended which might have introduced a response bias. As with any survey study, survey responses are limited by interpretation of questions and are vulnerable to error. Our survey was collected anonymously, as a result, we are unable to determine the exact demographics of our responders. We are unable to acertain the percentage of respondents from tertiary care centers, small community centers, or the specific application of this data to different levels of health care centers. Additionally, our survey was only distributed to academic institutions which limits the generalizability of our findings. Finally, as with any survey, survey responses are limited by interpretation of questions and are vulnerable to error.

## Conclusion

Currently, there is a great deal of variability with regard to non-endocavitary, external transducer disinfection protocols at academic institutions in the United States. This variability seems to stem from the vast number of disinfectant products and ultrasound manufacturer disparate recommendations. In order to mitigate risk to patients and reduce healthcare costs linked to nosocomial infections, healthcare providers, ultrasound companies, and disinfectant manufacturers must develop a universal use disinfectant and a standard protocol for ultrasound device disinfection in the emergency department.

## Additional file

Additional file 1: Appendix A – Online Survey. (DOC 26 kb)
