# Supplementary material for: Ultrasound transducer disinfection in emergency medicine practice
Source: Antimicrob Resist Infect Control. 2016 Apr 4;5:12. doi: 10.1186/s13756-016-0110-y (PMC4820997; doi:10.1186/s13756-016-0110-y)
Supplement: Additional file 1: — Appendix A – Online Survey. (DOC 26 kb) [file 13756_2016_110_MOESM1_ESM.doc]

Appendix A – Online Survey

We are conducting a very brief survey regarding external-use transducer cleaning protocols. (This survey is not regarding the cleaning practices for intracavitary transducers.) There are differing recommendations between manufacturers and institutions regarding the cleaning of external-use transducers. As with other institutions, we need to communicate with our Infection Prevention Department and we seek to characterize what the current “Standard of Practice” is across the country. The results of our survey will be emailed directly to those who respond and enter their email address on the final survey question. There are only 5 questions and we anticipate this will take less than 3 minutes to complete.

1. Is it “Standard of Practice” at your institution to use soap (Liquinox) and water to remove gel from the external-use transducers (not intracavitary probes) prior to disinfecting the probe after every use? If you use other methods for cleaning, please specify in Other.

Yes 
No
 Other (please specify)

2. Is it “Standard of Practice” at your institution to use disinfectant solution or disinfectant wipes to disinfect the external-use transducers (not intracavitary probes) and its components after every use? If you use other methods for cleaning, please specify in Other.

Yes 
No
 Other (please specify)

3. What do you use to disinfect the external-use transducers (not intracavitary probes) after every use?

T-spray solution
PDI Sani-Cloth Plus wipes (RED TOP) 
PDI Super Sani-Cloth wipes (PURPLE TOP)
PDI Sani-Cloth HB wipes (GREEN TOP)

Other (please specify)

4. What is your “Standard of Practice” recommended disinfectant (Sani-Cloth or T-spray) duration of contact with the transducer (not intracavitary probe)?

Brief wipe down transducer (less than 15 seconds)
1-minute contact time
 2-minute contact time
3-minute contact time
Other (please specify)

5. Finally, if you would be willing to share your institution’s policy and other information, please email us directly at EMUS@aemrc.arizona.edu. Our questions specifically are as follows: Do you have any additional cleaning process, which is used on a recurring basis? Do you have a written policy in place for the cleaning process? How was your cleaning process developed (hospital policy, emergency department policy, informal practice, etc.)? Is your cleaning process different between machine brands?

Comments Box:
